# Expanded Gram-Negative Activity of Marinopyrrole A

**DOI:** 10.3390/pathogens14030290

**Published:** 2025-03-16

**Authors:** Clare F. Euteneuer, Brianna N. Davis, LeeAnna M. Lui, Andrew J. Neville, Paul H. Davis

**Affiliations:** Department of Biology, University of Nebraska at Omaha, Omaha, NE 68182, USA; ceuteneuer@unomaha.edu (C.F.E.); briannadavis@unomaha.edu (B.N.D.); leeanna.lui@einsteinmed.edu (L.M.L.); aneville@unomaha.edu (A.J.N.)

**Keywords:** antibacterial, marinopyrrole A, bacterial infections, susceptibility screening

## Abstract

The rise of bacterial infections is a global health issue that calls for the development and availability of additional antimicrobial agents. Known for its in vitro effects on Gram-positive organisms, the drug-like small molecule marinopyrrole A was re-examined for the potential of broader efficacy against a wider array of microbes. We uncovered selective efficacy against an important subset of Gram-negative bacteria from three genera: *Neisseria*, *Moraxella*, and *Campylobacter*. This susceptibility is correlated with the absence of canonical LPS in these specific Gram-negative species, a phenomenon observed with other hydrophobic anti-microbial compounds. Further, when exposed to molecules which inhibit the LpxC enzyme of the LPS synthesis pathway, previously resistant LPS-producing Gram-negative bacteria showed increased susceptibility to marinopyrrole A. These results demonstrate marinopyrrole A’s efficacy against a broader range of Gram-negative bacteria than previously known, including *N. gonorrhea*, a species identified as a priority pathogen by the WHO.

## 1. Background

An estimated 7.7 million deaths per year worldwide are related to bacterial infections, according to the recent Global Burden of Disease Study, making bacterial infections the second-largest global cause of death [1]. The world’s ever-changing ecosystem and the increase in globalization further bolster their impact on human health [2]. To combat this issue, a robust arsenal of clinically available antibiotics can help against these disease-causing bacteria. However, the current arsenal has several limitations that may prevent effective treatment. A primary limitation is due to antimicrobial resistance (AMR), as bacteria can evade current antimicrobial therapy, making some treatments ineffective and obsolete. Other limitations of antimicrobials include allergic and adverse side effects or having rigorous storage or administration requirements [3,4]. Ultimately, despite the hundreds of available clinical antibiotics, there is still an urgent need for new, effective antibiotics.

One product derived from marine *Streptomyces* strain CNQ-418 is a 1,3-bipyrrole pharmacophore designated marinopyrrole A [5]. Actinomycetes produce very chemically diverse metabolites and have been a source of novel bioactive compounds; the strain CNQ-418 was obtained from sediment samples near La Jolla, CA, and was surmised to be a new species of *Streptomyces* [5]. The complete culture extract of CNQ-418 demonstrated antibiotic activity upon preliminary testing, leading to more specific investigations into the metabolites to identify potential small molecules with antibiotic potentials—especially the marinopyrroles [5].

Marinopyrrole A has shown growth inhibition against certain human cancer lines and bacteria. The mechanism of action of marinopyrrole A is not completely defined. However, one suspected mode of action is against the mammalian anti-apoptotic myeloid cell leukemia cell differentiation protein, Mcl-1, which prevents apoptosis when highly expressed [6]. Common in many human cancer cell lines, closely related analogs have also been identified in other mammals and yeast [7]. In multiple studies, Maritoclax (marinopyrrole A) has been found to selectively inhibit Mcl-1 [7]. Maritoclax is a synthetic marinopyrrole A composed of a racemic mixture of two enantiomers rather than the natural (-) enantiomer [8,9]. The racemic mixture and (-) enantiomer have both been tested in cancer studies, with comparable potency; similar results were also found when evaluated against MRSA [10,11]. When used in conjunction with ABT-737, a mimetic to the pro-apoptotic protein BH3, Maritoclax overcame the overexpression of Mcl-1 and ABT-737 resistance [7]. In murine models with xenografted U937 tumors, 59.1% of tumors responded to Maritoclax treatment with significantly decreased tumor sizes overall with no toxicity effects [12].

Inhibitory activity is not limited to mammalian cancer cells; indeed, marinopyrrole A has been shown to be relatively effective towards a wide range of Gram-positive bacteria, including various drug-resistant strains of *Staphylococcus* [13,14]. *Staphylococcus aureus* and *Staphylococcus epidermidis* were both susceptible to the racemic mixture compound in vitro, as were the methicillin-resistant strains of each species (MRSA and MRSE) [14]. These findings suggest that the (-) and racemic mixture marinopyrrole A and related molecules could be a potential compound class effective against drug-resistant bacteria.

Gram-negative bacteria are frequently more resistant to antibiotics, often due to the presence of an additional outer membrane (OM) with multiple complex elements that work to prevent the entrance of many antibiotics. Antibiotics often enter bacterial cells by diffusion through the membrane or through porins, but Gram-negative bacteria can alter these elements, leading to resistance [15]. Mutations in chromosomal genes in Gram-negative bacteria can lead to the expression of these different resistance mechanisms, including changes in porins, antibiotic-inactivating pathways, efflux pump activation, membrane permeability changes, and others [15]. Also, while Gram-positive bacteria have one lipid membrane and a thick layer of peptidoglycan that can be 20–80 nm, Gram-negative bacteria have more complex composite consisting of a thin layer of peptidoglycan sandwiched between two lipid membranes [16]. Hydrophobic molecules can pass through the hydrophobic regions of the phospholipid bilayer of the Gram-positive bacteria without much hindrance from the peptidoglycan layer [17]. Whereas in Gram-negative bacteria, OM lipoproteins maintain the membrane integrity by preventing entrance of many large molecules, only allowing the diffusion of small hydrophilic molecules useful in downstream metabolism via β-barrel porins [18]. Additionally, lipopolysaccharides (LPS) are found on the outer leaflet of this exterior membrane, and their tight packing causes low permeability of the membrane while their outer-most components, hydrophilic oligosaccharides, sterically and chemically inhibit the entry of large and hydrophobic molecules [18]. Therefore, the efficacy of marinopyrrole A, a relatively large hydrophobic molecule with a molecular weight of 510.15, is expected to have less penetrance through this complex Gram-negative cell wall compared to Gram-positive bacteria.

For all but one previously screened Gram-negative bacteria species, marinopyrrole A was ineffective. However, *Haemophilus influenzae* serotype B [ATCC 10211, listed in [13] as 1021], a Gram-negative bacterium that causes infections such as pneumonia or meningitis in children, was found to be sensitive to marinopyrrole A with an MIC of 2 mg/L [13]. This study suggested that *H. influenzae* could be an anomaly as the other two Gram-negative bacteria evaluated had no susceptibility to marinopyrrole A. Another possibility is that there is a subset of Gram-negative organisms not previously known to be susceptible. Given the potential for expanded activity against select Gram-negative organisms, the objective of this study was to evaluate the in vitro efficacy of marinopyrrole A against a broader range of Gram-negative bacteria. Through this, we found that an important group of Gram-negative bacteria demonstrated susceptibility to marinopyrrole A in vitro.

## 2. Material and Methods

### 2.1. Bacterial Strains and Culture Conditions

A diverse spread of bacteria, Gram-negative and Gram-positive cocci and bacilli were tested to determine the in vitro antibacterial effects of marinopyrrole A. Gram-positive bacteria include *Staphylococcus epidermidis* strain BCM0060 [BEI HM-140] and *Enterococcus durans* 23C2 [ATCC 6056]. Eight Gram-negative bacteria include *Neisseria mucosa* strain C1202 [BEI HM-242], *Neisseria meningitides* M107 [ATCC 13077], *Neisseria gonorrhoeae* CDC Ng-90 [ATCC 43070], *Proteus mirabilis* strain WGLW4 [BEI HM-752], *Escherichia coli* strain Seattle 1946 [ATCC 25922], *Campylobacter jejuni* doylei 093 [BEI NR-124], *Moraxella catarrhalis* Ne 11 [ATCC 25238], and *Haemophilus influenzae* [ATCC 49247].

All bacteria were inoculated on an agar (Fisher Scientific, Fair Lawn, NJ, USA) plate of their respective media listed below, and then inoculated into liquid culture in their respective media. *S. epidermidis* was grown in Miller’s Luria–Bertani (LB) Broth (Fisher Scientific, Fair Lawn, NJ, USA, cat. BP9723-17-3) [19]. *P. mirabilis* was grown in Mueller Hinton Broth (Sigma-Aldrich, St. Louis, MO, USA; cat. 70192-500G) [20]. *N. mucosa*, *N. meningitides*, *N. gonorrhoeae*, *E. coli* and *E. durans* were grown in Tryptic Soy broth (Fisher Scientific, Fair Lawn, NJ, USA; cat. DF0370-17-3) [21,22,23,24]. *C. jejuni* was grown in Tryptic Soy Broth under microaerophilic conditions [25]. *M. catarrhalis* was grown in 5% CO_2_ in Brain–Heart Infusion broth (ThermoFisher Scientific, Waltham, MA, USA; cat. CM1135B) [26]. *H. influenzae* was grown in Brain–Heart Infusion broth (ThermoFisher Scientific, Waltham, MA, USA; cat. CM1135B) supplemented with hemin (Sigma-Aldrich, St. Louis, MO, USA; cat. 51820) and Nicotinamide adenine dinucleotide (A. G. Scientific, San Diego, CA, USA; cat. N-2645-1GM) [27]. All bacteria were grown at 35 °C and were shaken at 250 rpm.

### 2.2. Antimicrobial Agents and Assays

The racemic form of marinopyrrole A (Sigma-Aldrich, St. Louis, MO, USA; cat. SML1533) was received and stored as a solid at −20 °C until use. Stock solutions of 10,000 mg/L or 20,000 mg/L of synthetic (±)-marinopyrrole A were prepared by dissolving in 100% (*v*/*v*) dimethyl sulfoxide (DMSO; Sigma-Aldrich, St. Louis, MO, USA; cat. D8418) and stored as aliquots at −80 °C. Gentamicin sulfate (Corning, Corning, NY, USA; cat. 30-005-CR) was stored at 2 °C until use.

Antimicrobial susceptibility testing was done by broth microdilution assays performed in clear non-treated 96-well round bottom microplates (Corning, Corning, NY, USA; cat. 351177). Marinopyrrole A was added to wells with a three-fold serial dilution (50–0.2 mg/L) with each concentration performed in triplicate and gentamicin in duplicate (50–0.05 mg/L). All tested compound concentrations were standardized to have a final concentration of 0.5% (*v*/*v*) DMSO, with each compound freshly diluted in solvent and its respective broth at the start of each assay. Fastidious organisms (*C. jejuni*, *N. gonorrhoeae*, *N. meningitidis*, and *H. influenzae*) were grown for 48 h, and all other organisms were grown for 24 h; then, all were diluted to an OD_600_ value of 0.05 and were delivered to the plate. The final volume was 100 µL/well. Non-fastidious bacteria were compound-treated for 24 h in 35 °C while shaking at 250 rpm, while fastidious organisms were grown for 48 h under the same conditions along with 5% CO_2_. OD_600_ values were used to determine average percent optical density and growth inhibition and were measured using a Biotek Synergy HT microplate reader. The minimum inhibitory concentration was determined by the lowest concentration with no visible growth. The half-maximal inhibitory concentration (IC_50_) and 90% inhibitory concentration (IC_90_) were determined through the hillslope formula, shown below, and Graphpad’s EC_anything_ calculator, respectively, as compared to control wells with 0.5% DMSO [28,29]. Bacteria exhibiting MIC and IC_50_ ≤ 2 mg/L were considered susceptible [30].

Hill slope: Y = BOTTOM + (TOP − BOTTOM)/(1 + 10 ^ ((LOG IC50 − LOG X) × SLOPE))

### 2.3. LpxC Inhibitor Assay

Antimicrobial susceptibility testing was performed on *P. mirabilis* by broth microdilution assays with and without LpxC inhibitor molecules. Log-phase *P. mirabilis* organisms were diluted to an initial OD_600_ value of 0.05 and then added to 96-well plates. Two LpxC inhibitors, CHIR-090 (Sigma-Aldrich, St. Louis, MO, USA; cat. SML3092) or PF-04753299 (Sigma-Aldrich, St. Louis, MO, USA; cat. PZ0284), were then added to plates at MIC_90_ and sub-MIC_90_ values and incubated for an hour before addition of marinopyrrole A or gentamicin. CHIR-090 had final concentrations of 0.0025, 0.025, and 0.25 mg/L, while PF-04753299 had final concentrations of 0.02, 0.2, and 2 mg/L [31,32]. After 1 h of incubation with LpxC inhibitor, each sample was duplicated with final concentrations of 20–0.2 mg/L for marinopyrrole A and 50–0.05 mg/L for gentamicin and were incubated and evaluated as above. Statistical analysis was run between growth of solvent only treated controls and growth of bacteria only exposed to LpxC inhibitors. An unpaired, two-sample equal variance two-tailed Student *t*-test was run with an alpha of 0.05.

## 3. Results and Discussion

Historically, marinopyrrole A has been known to inhibit Gram-positive bacterial growth at MICs often below 2 mg/L, as briefly reviewed in Table 1; whereas Gram-negative organisms were often quite resistant, even beyond 50 mg/L in vitro [5,11,13,14,30,33].

Our own in vitro findings confirmed that marinopyrrole A produced low or sub-micromolar potency as well as high selectivity indexes among the tested gram-positive bacteria *Enterococcus durans* and *Staphylococcus epidermidis* (Table 2) when compared to cytotoxicity in non-cancerous human fibroblast cells [34]. Further, we observed that the Gram-negative bacteria *Proteus mirabilis* and *Escherichia coli* remained resistant with MICs greater than 50 mg/L, in line with past findings (Table 1) [13,14].

Previous experiments by others have documented that most Gram-negative bacteria are resistant to marinopyrrole A as well as its derivatives [13,14,33]. Notably, the bacteria *Haemophilus influenzae* is an exception as it was reported by one group to be susceptible to marinopyrrole A [13]. We reasoned that the efficacy of marinopyrrole A against this particular Gram-negative organism could be related to the lipoglycan structure found in this species that is not shared by most previously tested Gram-negative bacteria. While lipopolysaccharide (LPS) is a common outer membrane lipoglycan shared among many Gram-negative bacteria, its structural components can have significant variations between bacterial species and even strains [18,35]. Most commonly, LPS is composed of three core elements, from inner to exterior order, Lipid A, a core oligosaccharide, and finally the O-antigen. Lipid A is a hydrophobic region that anchors the LPS to the outer membrane, while the core oligosaccharide contains hexoses, heptoses, and Kdo residues with differing modifications and connects Lipid A to the O-antigen [18]. The Lipid A and core oligosaccharide structures are generally conserved within species [18,36]. The O-antigen, however, is the most variable component, being a polysaccharide often containing 2–8 sugars and varying between strains of a bacterial species. However, some Gram-negative organisms, like *H. influenzae*, produce lipooligosaccharides (LOS) in place of LPS, while, similar to LPS, the lipoglycan LOS lacks the O-antigen, and has also been known historically as rough LPS [13,18,35,36,37]. Variation in the O-antigen, core, and Lipid A also result in the following classifications: smooth LPS with all components, semi-rough LPS with only one sugar of the O-antigen, Ra-LPS (rough LPS, LOS), and Re-LPS that lacks parts of the core [35]. The continuum of varying LPS structural variation among gram-negative bacteria, particularly pathogenic species, complicates efforts to elucidate its effects due to the strain-to-strain differences, and dynamic changes in response to environmental conditions [38].

Notably, all of the Gram-negative bacteria previously evaluated for marinopyrrole A susceptibility in past studies, besides *H. influenzae*, are known to produce LPS, not LOS. To explore the potential that marinopyrrole A could be effective against Gram-negative organisms producing LOS in place of LPS, two canonically known LOS-synthesizing bacteria were evaluated, *Campylobacter jejuni* and *Moraxella catarrhalis*, and both were found to be sensitive to marinopyrrole A (Table 3), comparable to Gram-positive sensitivity [36,37]. *C. jejuni* had an MIC of 2 mg/L and an IC_50_ of 0.74 mg/L, while *M. catarrhalis* had a MIC of 0.6 mg/L and an IC50 of 0.38 mg/L. These bacteria have not previously been evaluated for marinopyrrole A sensitivity and represent a new potential therapeutic against these and related pathogens.

*Campylobacter jejuni* is the leading cause of bacterial diarrhea cases in the United States, with an estimated 1.5 million cases a year [39]. It is contracted most often from raw or undercooked poultry or exposure to infected animals and has a very low infectious dose [40]. While most individuals can overcome infection without treatment, at-risk individuals such as the elderly or those with compromised immune systems can require treatment to prevent complications [41]. While macrolides are the current treatment recommendation, the use of fluoroquinolones is common initially when the bacterial pathogen is unknown [39]. However, the use of fluoroquinolones in farm animals, specifically chickens, has led to widespread resistance, and some resistance to macrolides such as erythromycin has also been reported [39,42]. *Moraxella catarrhalis* was once considered a commensal organism residing in human respiratory tract but has been more recently identified as pathogenic and common in causing otitis media in children and chronic obstructive pulmonary disease in adults [43]. While infections can currently be treated by oral antibiotics such as newer macrolides and trimethoprim-sulfamethoxazole, above 90% of *M. catarrhalis* are resistant to ampicillin and further resistance to second-generation cephalosporins has been observed since 2000 [43,44].

In addition, we evaluated members of the *Neisseria* genus, which are Gram-negative bacteria also known to produce LOS in place of LPS [35,36,37,45]. Importantly, *Neisseria gonorrhoeae*, the causative pathogen of gonorrhea, is the second most common sexually transmitted bacterial infection globally and is on the high priority list of the World Health Organization Bacterial Priority Pathogens List for 2024 [21,46]. As an LOS-producing bacteria, we hypothesized that *N. gonorrhoeae* would have susceptibility similar to that of *H. influenzae* and gram-positive bacteria for marinopyrrole A. Interestingly, *N. gonorrhoeae* was the most susceptible bacterium to all tested concentrations of marinopyrrole A, having a MIC less than 0.2 mg/L and an IC_50_ below 0.15 mg/L.

New cases of gonorrhea are estimated at 82.4 million globally [47]. While many cases are asymptomatic, the disease can lead to pelvic inflammatory disease, ectopic pregnancy, infertility, pre-term births, neonatal conjunctivitis leading to blindness, epididymitis in men, as well as a higher risk of contracting other STIs including HIV [21,32,46,48]. Due to the lack of a gonococcal vaccine and increasing rates of MDR strains of *N. gonorrhoeae* resistant to multiple classes of antibiotics, there is a serious need for drug exploration for this pathogen [46,48]. Current treatment recommendations include third-generation cephalosporins, macrolides, tetracyclines, trimethoprim combinations, and quinolones [49,50]. However, resistance to all these classes of drugs has been reported globally. In a global meta-analysis of reports until 2021, resistant samples across all countries were detected for ciprofloxacin at 52%, tetracycline at 45% (but up to 100% resistance in Africa), and TMP/SMX at 42% with the majority of resistance levels increasing over time [49].

*Neisseria meningitidis* is another pathogenic species of the *Neisseria* genus associated with invasive meningococcal disease that can lead to septicemia or meningitis [51,52,53]. While vaccines have been developed for most serogroups and drug resistance is not yet common, 5–10% of cases are fatal even with current antibiotic treatment [51,52]. These pathogenic and non-pathogenic species of *Neisseria* were also evaluated for sensitivity to marinopyrrole A. *N. meningitidis* had sensitivity to the compound at levels similar to gram-positive sensitivity with a MIC of 2 mg/L and an IC_50_ of 1.3 mg/L (Table 3).

To further investigate the potential susceptibility differences due to lipoglycan structures in gram-negative organisms, synthetic marinopyrrole A was co-administered with either of two unique LpxC enzyme inhibitor molecules. This approach was used to evaluate marinopyrrole A sensitivity when the presence of canonical LPS structures is reduced. CHIR-090 is capable of interfering with the production of LPS by inhibition of LpxC, the second enzyme in the Lipid A synthesis pathway, causing LPS levels to be significantly reduced in other evaluated bacteria [31,54]. CHIR-090 is a slow, tight-binding LpxC inhibitor that has been shown to have effects on LpxC homologs in various gram-negative bacteria. CHIR-090 has a hydroxamic acid group that binds zinc in the LpxC active site [31]. It also has a hydrophobic tail that resembles the LpxC enzyme substrate hydroxy-myristate’s fatty acid group, allowing weak interactions with the hydrophobic tunnel found in the active site [31]. Historically, high doses of CHIR-090 between 0.25 and 40 mg/L can achieve an MIC_90_ against various Gram-negative bacteria [31]. Thus, when co-administered with marinopyrrole A, the lowest MIC_90_ value (0.25 mg/L) as well as sub-MIC values corresponding to 1/10 and 1/100 of previously established MIC_90_ values were used to inhibit LPS production while reducing the potential of overall membrane integrity disruption. Similar approaches with this compound have been used to increase susceptibility to antimicrobial agents such as vancomycin and rifampin [55].

**Figure 1:**
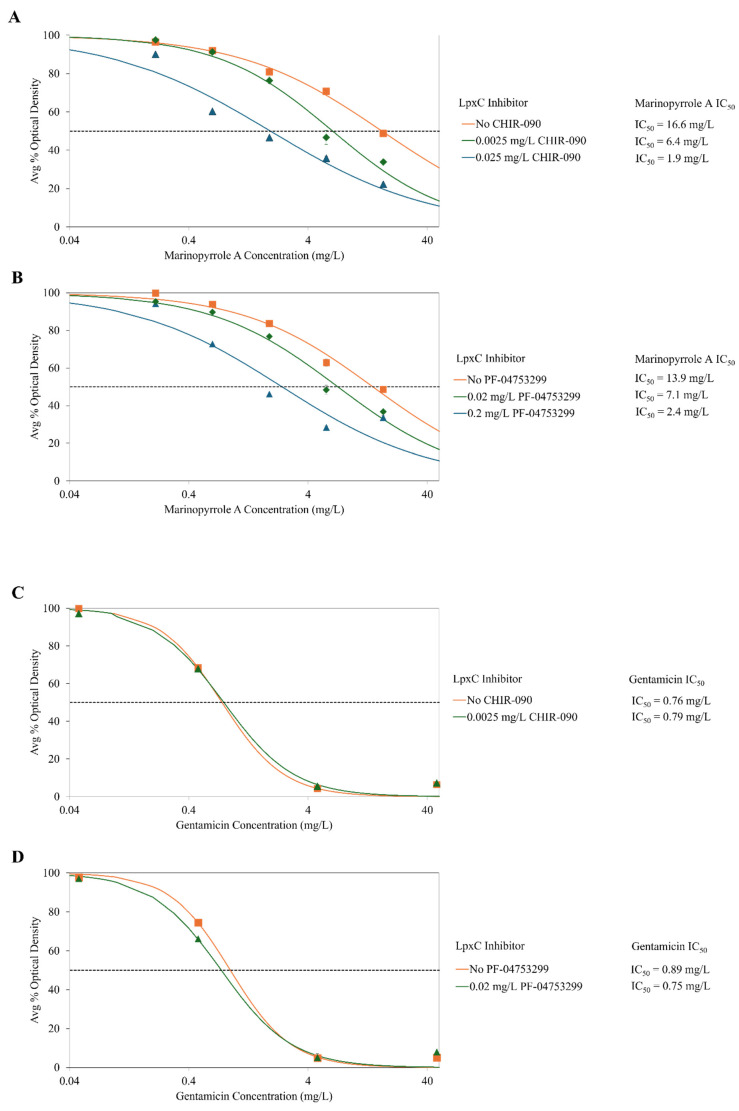
Susceptibility (IC_50_) of gram-negative bacteria *P. mirabilis* to marinopyrrole A and gentamicin when co-treated with CHIR-090 or PF-04753299, both LpxC enzyme inhibitors. CHIR-090 and PF-04753299 are both competitive inhibitors for LpxC, an enzyme essential to the production of LPS. These inhibitors can cause reductions in LPS synthesis in Gram-negative organisms. When marinopyrrole A was co-treated with CHIR-090 (**A**) or PF-04753299 (**B**), the inhibitors caused a dose-dependent reduction in LPS synthesis which led to an increase in marinopyrrole A susceptibility of *P. mirabilis* (canonical LPS-producing Gram-negative bacteria). The potency of the positive control compound gentamicin is not affected in the presence of LpxC inhibitors (**C**,**D**). Average percent bacterial density was calculated using OD_600_ measurements after 24 h of incubation. IC_50_s were calculated with hill-slope modeling by normalizing to the average percent optical density of solvent-only controls exposed to the corresponding concentration of LpxC inhibitor.

**Figure 2:**
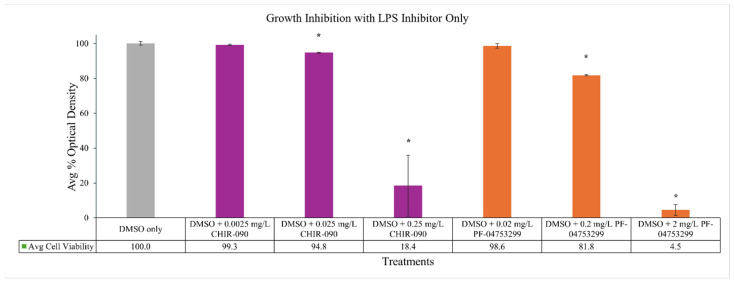
Inhibition of bacteria *Proteus mirabilis* treated with LpxC enzyme inhibitors alone. Inhibition of bacteria is not impacted by treatment with LpxC inhibitors alone: optical density remains above 90% for 0.0025 and 0.025 mg/L of CHIR-090, and above 80% for 0.02 and 0.2 mg/L of PF-04753299. Average percent bacterial density calculated using OD600 measurements after 24 h of incubation. Statistical analysis done by unpaired student’s *t*-test, * denotes *p*-values < 0.001 when compared to DMSO only.

As the Gram-negative bacterium *P. mirabilis* is less susceptible to marinopyrrole A with its high IC_50_ of 17 mg/L, and is known to produce a canonical LPS structure, it was selected for LpxC inhibition studies along with co-administration of marinopyrrole A. When co-administered with CHIR-090, the susceptibility of normally LPS-producing *P. mirabilis* to marinopyrrole A increased in a dose-dependent manner, shown by decreases in the IC_50_ values (Figure 1): exposure to CHIR-090 at 0.0025 mg/L and 0.025 mg/L had decreased marinopyrrole A IC_50_ values by 2.6 and 8.7-fold, respectively, when compared to the IC_50_ of marinopyrrole A alone. Importantly, inhibitor alone at these exposure levels were found to be 99% and 95%, as compared to no inhibitor (Figure 2). Moreover, susceptibility to gentamicin did not change with 0.0025 mg/L CHIR-090, suggesting that the membrane integrity of the bacteria was still intact when exposed to these sub-MIC concentrations of CHIR-090 (Figure 1). Altogether, this suggests that decreasing the LPS synthesis through LpxC inhibition led to greater susceptibility of this Gram-negative organism to marinopyrrole A.

Growth of the bacteria with sub-MIC levels of solely the inhibitor at 0.0025 and 0.025 mg/L were found to be 99% and 95% the level of growth as compared to no inhibitor (Figure 2), supporting the hypothesis that partial loss of LPS allows marinopyrrole A to become effective against this normally resistant Gram-negative bacteria. 

A second LpxC enzyme inhibitor, PF-04753299, is a methyl sulfone compound that also can interfere with LPS synthesis, most particularly in *E. coli*, but also has demonstrated inhibitory effects in other Gram-negative bacteria [31]. PF-04753299 also has hydroxamic acid zinc-binding group, a shorter hydrophobic tail that fit into the LpxC active site, and hydrogen bonding interactions between the alkyl sulfone headgroup and lysine residue near the active site [31]. This compound also has been used in the past at sub-MIC levels to increase susceptibility of gram-negative bacteria to antimicrobial agents such as vancomycin and rifampin, but to a lesser extent than CHIR-090 [55]. As the MIC_90_ value was reportedly 2 mg/L, 1/10 and 1/100 of the MIC_90_ were used to measure changes in inhibitory concentrations [31]. As PF-04753299 concentration was increased, the susceptibility of LPS-producing *P. mirabilis* to marinopyrrole A similarly increased when in the presence of 0.02 and 0.2 mg/L of PF-04753299, the IC_50_s of marinopyrrole A against LPS-producing *P. mirabilis* to marinopyrrole A decreased by 2.0- and 5.8-fold, respectively. Growth of the bacteria with sub-MIC levels of PF-04753299 alone at 0.0025 and 0.025 mg/L was found to be 99% and 82% respectively as compared to no inhibitor (Figure 2). Once again, the susceptibility of P. mirabilis to gentamicin had minimal differences between no inhibitor and 0.02 mg/L PF-04753299 (Figure 1).

Results from two independent LpxC inhibitors, known to be essential for LPS synthesis, suggest that the reduction of canonical LPS-structures in Gram-negative bacteria increases susceptibility to marinopyrrole A. The application of two independent LpxC compounds reduces the likelihood of strong synergistic activity with marinopyrrole A unrelated to LPS synthesis; nor have any LPS-unrelated synergistic effects been reported with these two inhibitors.

As suggested by augmented effects of marinopyrrole A on bacteria with LPS reduction, LPS is a major component in the resistance of gram-negative bacteria to antibacterial compounds. The outer leaflet of the OM of Gram-negative bacteria includes LPS instead of solely a phospholipid bilayer, allowing more resistance to hydrophobic molecules that would normally pass through a phospholipid bilayer [18,56]. This barrier effect is mostly due to the low fluidity of the membrane and the hydrophilic exterior components of LPS. The fatty acid chains of the Lipid A section are saturated and hydrophobic, which allows for considerable interactions between chains and results in low membrane fluidity. The Lipid A and core oligosaccharide of LPS have multiple negatively charged phosphate groups, which would normally limit tight packing; however, the presence of divalent cations such as Ca^2+^ and Mg^2+^ allows for the negatively charged phosphate groups to be engaged in polyionic interactions. This leads to stronger lateral interactions between LPS molecules than exist between phospholipid molecules, increasing barrier function [18]. The core oligosaccharide and the O-antigen particularly have hydrophilic properties that disrupt the passage of hydrophobic molecules [18,56]. The O-antigen component of the LPS has been shown to provide resistance of the bacteria to detergents and hydrophobic molecules [57]. LOS bacteria have been seen to be more sensitive to such molecules as their membranes are more hydrophobic without the increased (poly)saccharides regions and we theorize provide favorable environments to allow for hydrophobic molecules to interact and/or pass the outer membrane.

Marinopyrrole A is not the first molecule to have differential growth inhibitory effects on LOS-producing Gram-negative bacteria. Previous studies have found that highly hydrophobic molecules, such as macrolides and rifampin, have variable results across gram-negative bacteria with membrane lipopolysaccharides with varying lengths, compositions, and presence (LPS) or absence of (LOS) O-antigen structures [13,58,59]. The lack of O-antigens further reduces the steric hindrance and given its mostly polar and hydrophilic properties, also plays a role in the susceptibility of marinopyrrole A, as well as other molecules with similar physicochemical properties (pyrrolomycins). Marinopyrrole A has a predicted logP value of 6.11, which is considered quite hydrophobic [60]. The results from our experiments suggest that the hydrophobicity of marinopyrrole A and its inhibitory effect upon LOS-expressing gram-negative bacteria is likely due to lack of the O-antigen in the outer membrane matrix of these bacteria.

This idea is also reflected in the antibiotic rifampin, another hydrophobic compound, that has efficacy against Gram-negative LOS bacteria, including *H. influenzae*, *N. meningitidis*, and others [53]. Rifampin is a larger molecule than marinopyrrole A, at 822.9 g/mol, and has similar MICs against LOS bacteria to marinopyrrole A [61,62]. For example, the rifampin MIC for *H. influenzae* is 0.5–0.64 mg/L, as compared to the MIC of 0.6 mg/L for marinopyrrole A. Similarly, the MIC of rifampin for *N. gonorrhoeae* was found to be 0.06–2 mg/L, which is very similar to that found for marinopyrrole A at <0.2 mg/L [53]. The MICs for rifampin on LPS Gram-negative bacteria were comparable to that of marinopyrrole A, with higher MICs of 25 mg/L for *E. coli* and up to 64 mg/L for *P. mirabilis* [63,64]. While these two have similar MICs, rifampin has been found to have resistance incurred against it by these bacteria, leading to the need for a novel, similarly effective, compound [61].

More recent work has been done to further understand how marinopyrrole A may act against Gram-positive bacteria as a protonophore [33]. Marinopyrrole A and related compounds (pyrrolomycins) have been shown to act as protonophores that cause the depolarization of bacterial membranes and impact the proton motor force by reducing the protein-coupled transport of protons [33,65]. The import and export of protons is essential in many bacterial processes, including ATP synthesis and the active transport of metabolites inside the cell [65]. The acidic phenyl hydroxyl and pyrrole N-H groups present in these compounds, both with low pKa, can participate in the shuttling of protons across the membrane, depositing them in the cytoplasm [33,65]. The outer membrane of Gram-negative LOS bacteria is likely less polar (having less saccharides) and enables a closer proximity of the molecule to the membrane. As the steric and electrostatic hindrance is supposedly decreased because of the lack of the O-antigen polysaccharides, this may account for these specific Gram-negative bacteria being more susceptible to the hydrophobic marinopyrrole A and its protonophore activity.

It has been suggested that marinopyrrole A acts in this way as a proton shuttle that can move protons across the membrane and therefore disrupt the proton motive force and membrane potential of bacterial cells [33]. Notably, marinopyrrole A derivatives with greater acidity and greater hydrophobicity have higher antibacterial activity potentially due to their stronger affinity for the membrane environment, suggesting that these derivatives may be a valuable area of additional exploration [33]. Along with this, due to the small size of marinopyrrole A, it is possible that a combination of potential protonophore action and free migration could be possible modes of entry into bacterial cells [33].

While marinopyrrole A has excellent activity against Gram-positive bacteria such as MRSA and now an important subset of Gram-negative bacteria, its activity in vitro has been shown to be reduced in the presence of human serum [13]. It was found that its effect against MRSA was effectively neutralized by the presence of 20% human serum in vitro [13]. While this finding may impact its capabilities in vivo, marinopyrrole A has displayed increased survival time and improved mortality when administered to mice after a lethal infection of *Toxoplasma gondii* [34]. Additionally, it has been suggested to potentially serve as a topical agent, which would avoid concerns about inactivation by human serum [11]. Furthermore, to increase clinical application, promising new derivatives designed with decreased sensitivity to human serum and reduced cytotoxicity will be valuable to future efforts [34]. These derivatives were designed using the fragment-based approach and have been found to have no cytotoxicity in HFF cells tested up to 25 mg/L [34]. Along with this, when tested in vitro for serum sensitivity, they were found to have less sensitivity than marinopyrrole A and retained potency up to 50% serum [34]. Such derivatives constitute an additional avenue of study that could lead to potential clinical relevance for this class of antibiotics.

## 4. Conclusions

We sought to further evaluate the antibacterial spectrum of activity of the drug-like compound marinopyrrole A across a wider array of Gram-negative bacteria than had been previously investigated. Our findings demonstrate activity against subset of at least five gram-negative bacteria strains from three distinct genera found to be susceptible to marinopyrrole A at levels similar to those of gram-positive organisms. This subset of gram-negative bacteria shares similar cell wall architecture, namely those expressing the lipoglycan LOS instead of LPS on their outer membrane. We further suggested that a Gram-negative bacterium was made susceptible to marinopyrrole A when exposed to either of two LpxC enzyme inhibitors, suggesting that a decrease in the LPS production led to a decrease in the resistance mechanism of the bacteria against this compound.

Further work is needed to test this as well as an ever-broader range of Gram-negative organisms with both marinopyrrole A and its future derivatives. Along with this, more work is needed to investigate the susceptibility of multi-drug resistant (MDR) strains of LOS-producing bacteria, such as MDR *N. gonorrhoeae*, and whether the use of co-administered LPS disruptors would permit a much wider range of gram-negative organisms to become sensitive to marinopyrrole A and its derivatives. Despite toxicity and serum sensitivity issues observed in vitro, there is evidence of some efficacy in vivo using marinopyrrole A, and even more with synthesized derivatives [34]. Further development and testing of derivatives to address these issues may lead to promising candidates more suitable for preclinical in vivo studies and potentially future clinical indications.

## Figures and Tables

**Table 1:** Susceptibility (MIC) of bacteria against marinopyrrole A reported in previous literature. Marinopyrrole A inhibits growth among most Gram-positive and some Gram-negative bacteria.

Organism	MIC (mg/L)	References
Gram-positive Bacteria		
*Bacillus anthracis*	1–2	[13]
*Bacillus subtilis*	0.13–1.9	[13,33]
*Enterococcus faecalis*	1–6.8	[13,33]
Highly vancomycin-resistant *Enterococcus faecalis*	16–128	[14]
*Enterococcus faecium*	4	[33]
*Listeria ivanovii*	0.26	[33]
*Staphylococcus aureus*	0.5–1	[13,14,33]
Methicillin-resistant *Staphylococcus aureus*	0.2–1	[13,14,33]
*Staphylococcus epidermidis*	0.25–2.7	[13,33]
Methicillin-resistant *Staphylococcus epidermidis*	0.06–1	[14]
*Streptococcus agalactiae*	2	[13]
*Streptococcus pneumonia*	0.13	[33]
*Streptococcus pyogenes*	1	[13]
Gram-negative Bacteria		
*Acinetobacter baumannii*	>33	[33]
*Escherichia coli*	>33	[13,14,33]
*Haemophilus influenzae*	2	[13]
*Klebsiella aerogenes*	>33	[33]
*Klebsiella pneumoniae*	>16	[13,14,33]
*Ochrobactrum anthropi*	16	[33]
*Providencia alcalifaciens*	>33	[33]
*Pseudomonas aeruginosa*	>33	[13,14,33]
*Salmonella enterica*	>33	[33]
*Shigella sonnei*	>33	[33]
*Vibrio cholerae*	>33	[33]
*Yersinia pseudotuberculosis*	>33	[33]

**Table 2:** Susceptibility (MIC, IC_50_, and IC_90_) of bacteria against marinopyrrole A and gentamicin and Selectivity Index of marinopyrrole A with HFF. As described in the Methods, four bacteria species were tested in this study for sensitivity to marinopyrrole A. The Gram-negative *E. coli* and *P. mirabilis* strains tested here produce canonical LPS attached to their outer membrane, whereas *E. durans* and *S. epidermidis* are Gram-positive. MIC and IC_50_ values of ≤2 mg/L were considered susceptible [30].

Organism	Marinopyrrole A (mg/L)			Gentamicin (mg/L)			Marinopyrrole A Selectivity Index
	MIC	IC_50_	IC_90_	MIC	IC_50_	IC_90_	HFF IC_50_ (mg/L)/Bacteria IC_50_ (mg/L) *
*Enterococcus durans*	2	0.20	0.53	24	7.5	14.6	>125
*Staphylococcus epidermidis*	<0.2	<0.15	<0.15	48	3.0	7.3	>167
*Escherichia coli*	>50	>50	>50	2	1.3	18.8	0.5
*Proteus mirabilis*	>50	17	260	5	0.76	5.0	>1.5

* Human foreskin fibroblast (HFF) IC50 > 25 mg/L [34].

**Table 3:** Susceptibility (MIC, IC_50_, and IC_90_) of lipooligosaccharide (LOS) Gram-negative bacteria against marinopyrrole A and gentamicin. As described in the Methods, several Gram-negative bacteria species were evaluated for sensitivity to marinopyrrole A. Those bacteria listed here are known to produce the lipoglycan LOS in place of LPS on their outer membrane. They are notably sensitive to marinopyrrole A at potencies similar to those seen in gram-positive organisms. MIC and IC_50_ values of ≤2 mg/L were considered susceptible to marinopyrrole A [30].

Organism	Marinopyrrole A (mg/L)			Gentamicin (mg/L)		
	MIC	IC_50_	IC_90_	MIC	IC_50_	IC_90_
*Campylobacter jejuni*	2	0.74	6.7	0.2	0.09	0.1
*Moraxella catarrhalis*	0.6	0.17	1.5	5	0.2	0.6
*Haemophilus influenzae*	0.6	0.38	9.2	5	1.3	1.6
*Neisseria mucosa*	6	1.2	2.3	5	1.1	2.3
*Neisseria gonorrhoeae*	<0.2	<0.15	<0.15	2	0.8	0.9
*Neisseria meningitidis*	2	1.3	1.5	16	4.7	16.9

## Data Availability

Data are contained within the article.
